# Preparation of phospholipid-based polycarbonate urethanes for potential applications of blood-contacting implants

**DOI:** 10.1093/rb/rbaa037

**Published:** 2020-09-06

**Authors:** Peichuang Li, Wanhao Cai, Xin Li, Kebing Wang, Lei Zhou, Tianxue You, Rui Wang, Hang Chen, Yuancong Zhao, Jin Wang, Nan Huang

**Affiliations:** r1 Key Lab. of Advanced Technology for Materials of Education Ministry, Southwest Jiaotong University, Chengdu 610031, China; r2 School of Materials Science and Engineering, Southwest Jiaotong University, Chengdu 610031, China; r3 Institute of Physical Chemistry, University of Freiburg, Albertstraße 21a, Freiburg 79104, Germany

**Keywords:** phospholipid, polycarbonate urethanes, non-specific adhesion, anti-thrombosis, histocompatibility

## Abstract

Polyurethanes are widely used in interventional devices due to the excellent physicochemical property. However, non-specific adhesion and severe inflammatory response of ordinary polyurethanes may lead to severe complications of intravenous devices. Herein, a novel phospholipid-based polycarbonate urethanes (PCUs) were developed *via* two-step solution polymerization by direct synthesis based on functional raw materials. Furthermore, PCUs were coated on biomedical metal sheets to construct biomimetic anti-fouling surface. The results of stress–strain curves exhibited excellent tensile properties of PCUs films. Differential scanning calorimetry results indicated that the microphase separation of such PCUs polymers could be well regulated by adjusting the formulation of chain extender, leading to different biological response. *In vitro* blood compatibility tests including bovine serum albumin adsorption, fibrinogen adsorption and denaturation, platelet adhesion and whole-blood experiment showed superior performance in inhibition non-specific adhesion of PCUs samples. Endothelial cells and smooth muscle cells culture tests further revealed a good anti-cell adhesion ability. Finally, animal experiments including *ex vivo* blood circulation and subcutaneous inflammation animal experiments indicated a strong ability in anti-thrombosis and histocompatibility. These results high light the strong anti-adhesion property of phospholipid-based PCUs films, which may be applied to the blood-contacting implants such as intravenous catheter or antithrombotic surface in the future.

## Introduction

Non-specific adhesion or host response, is one of the most important problems in biological safety evaluation [1–5]. The host response is usually accompanied with acute inflammation, which may lead to severe complications of intravenous devices. In last few decades, various materials have been investigated for the biological applications [6–12]. Among them, polyurethanes (PUs) are regarded as a potential material in modern medical treatments because of the excellent mechanical property and special structure [11–14]. However, the host response problem is also widely observed with PUs, which strongly inhibit the practical application of PUs [15–17]. Thus, it is expected that optimal material design to prevent host response will be a promising strategy in this study of biomedical PUs.

To avoid non-specific adhesion of molecules and cells onto the device, surface with good anti-adhesion property is required. The introduction of functional molecules [1, 3–6, 18–21] has become one of the most important strategies for surface modification due to the optionality of raw materials. Up to now, immobilization of one kind of molecule and co-immobilization of more than two kinds of molecules have been reported in PUs surface modifications and great progress has been realized [22–28]. For example, the antibacterial and anticoagulant functions can be realized by introducing antibacterial molecules like quaternary ammonium salt [22, 23] and anticoagulation molecules like heparin [25, 26], respectively. Furthermore, multiple functions can be also realized *via* co-immobilization of bioactive molecules. However, some problems still remain unsolved. One important problem is that the unavoidable dissociation of functional molecules from the material with the time. Since the immobilization works only on the surface area, the dissociation will lead to a large decrease in the surface functions. Therefore, the long-term function stability can hardly be guaranteed in the application of PUs only with this method.

It is expected that this problem can be solved by direct synthesis based on functional raw materials [29–32]. Since the functional groups are integrated into the materials directly, this strategy could not only avoid the subsequent surface functionalization steps, but also ensure the long-term stable function of material surface. Since the 1980s, there have been extensive reports about the `–material interactions of polyurethanes and related systems. Speckhard *et al*. [33] has found that zwitterionization could impact the properties of polyurethanes, and possess higher thromboresistance. Besides, Yung and Cooper [34] also found that the incorporation of phosphorylcholine into the polyurethanes could effectively reduce neutrophil adhesion. Typical and efficient synthesis method of MPC was reported by Ishihara *et al.* [35] and their research team did a lot of works on the polymers containing 2-methacryloyloxyethyl phosphorylcholine (MPC) [36]. Moreover, they also proved that phosphorylcholine group could effectively reduce protein adsorption [37]. Zhang *et al*. prepared zwitterionic polyurethanes with free radical polymerization and polyaddition, and also found that the high content of polyurethanes can effectively resist non-specific protein adsorption [38]. More recently, Jiang *et al*. has extensively studied the incorporation of zwitterionic functionality into polymers and found superior non-fouling results [39–41]. Also, to better understand the mechanism of anti-fouling of zwitterionic component, the team of Jiang also explored the relationships between the unique properties of zwitterionic materials and their molecular structures with simulation and modeling studies [42]. Besides, the team of Jiang studied the incorporation of zwitterionic functionality into polyurethanes, such as the modification of segmented polyurethane with cross-linked sulfobetaine methacrylate polymer [43]. Moreover, Jiang *et al.* modified materials surface with zwitterionic carboxybetaine copolymers [44, 45] and fabricated tube of zwitterionic hydrogels [46]. These studies all revealed that zwitterionic component could effectively resist non-specific adhesion and shows great promise for blood-contacting devices.

In this study, to decrease non-specific adhesion and inflammatory response, a phospholipid-based polycarbonate urethanes (PCUs) is developed *via* two-step solution polymerization. Different from aforementioned modification methods including surface modification, physical blending and complicated process, this simple and feasible strategy could effectively change the surface and bulk properties. Moreover, the process of use is convenient and stable. The results of Fourier transform infrared spectroscopy (FTIR) and nuclear magnetic resonance (NMR) proved the successful synthesis of PCUs. The results of stress–strain curves exhibited excellent tensile properties of PCUs films. The results of atomic force microscope (AFM), X-ray photoelectron spectroscopy (XPS), scanning water contact angle (WCA) and differential scanning calorimetry (DSC) revealed the change of physical properties. Furthermore, PCUs were coated on 316L stainless steel (SS) sheets to construct biomimetic anti-fouling surface. A series experimental results including platelet adhesion, bovine serum albumin (BSA) adsorption, fibrinogen adsorption and whole-blood test indicated a strong ability of reducing non-specific adhesion and thrombosis. Endothelial cells (ECs) and smooth muscle cells (SMCs) culture tests further revealed the properties of reducing non-specific adhesion. Finally, animal experiments including *ex vivo* perfusion experiment and subcutaneous inflammation test indicated the remarkable abilities of anti-thrombosis and histocompatibility. These investigations high light the strong anti-adhesion property of phospholipid-based PCUs films, which may be applied to the blood-contacting devices such as intravenous catheter or antithrombotic surface in the future.

## Materials and methods

### Materials

SS sheets were purchased from Baoji Non-ferrous Metal Co., Ltd (Baoji, China). MPC was obtained from the Nanjing Joy-Nature Technology Institute (Nanjing, China). 4,4′-Diphenylmethane-diisocyanate (MDI), BSA, sodium dodecyl sulfonate (SDS) and fibrinogen from human plasma were all purchased from Sigma-Aldrich. Polycarbonate-diol (PCDL) was received from UBE industries, LTD. 3-Mercapto-1,2-propanediol (MP) and diisopropylamine (DIPA) were obtained from TCI. Dimethylacetamide (DMAc) and 1,4-Butylene glycol (BDO) were distilled under vacuum before use. Rabbit Anti-human Fibrinogen γ chain antibody (1 mg/ml), Goat Anti-rabbit IgG/HRP (2 mg/ml) and Rabbit Anti-human Fibrinogen/HRP-conjugated antibody were purchased from Bioss. All other reagents were analytical grade and purchased from Kelong Chemical Reagent Co., Ltd (Chengdu, China).

### Synthesis of MPCDL and PCUs

**Figure 1.**
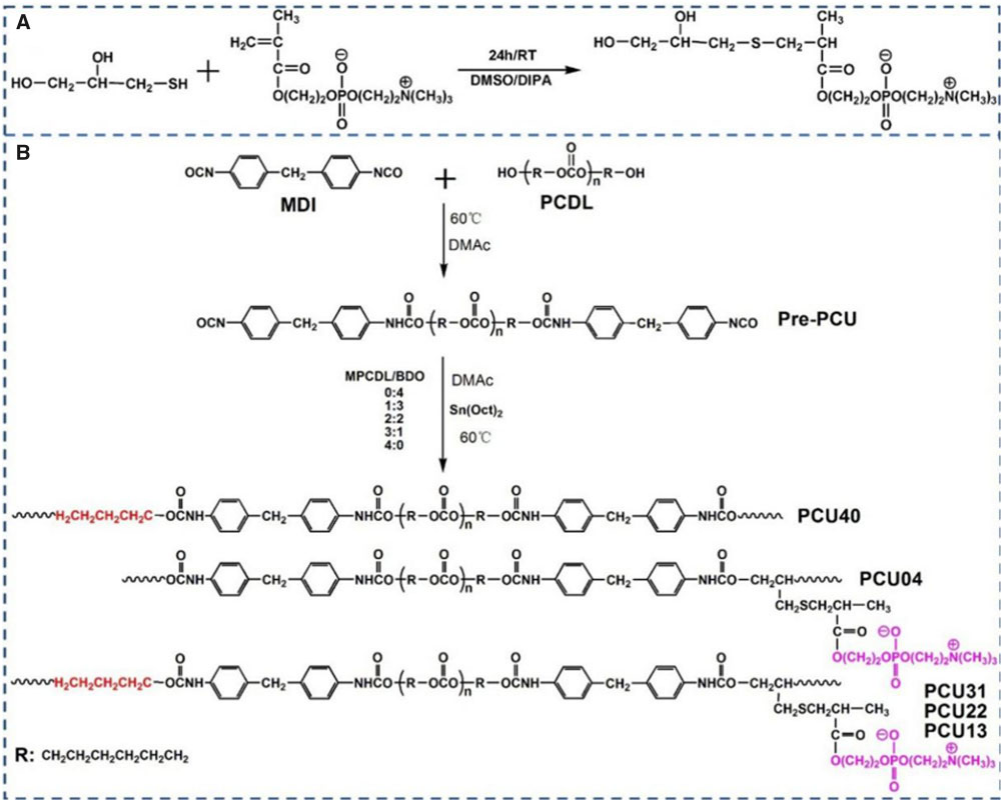
The synthesis route of (**A**) MPCDL and (**B**) PCU40, PCU31, PCU22, PCU13 and PCU04

As shown in Fig. 1A, MPCDL was synthesized *via* simple addition reaction between MPC and MP. First, MPC (10 mmol) and MP (10.2 mmol) were introduced into a round-bottomed flask under argon atmosphere. Then, DIPA (0.25 mmol) was added to the solution as a catalyst. After stirring at 25°C overnight, the product was precipitated from the reaction solution *via* cold diethyl ether. After that, the product was purified by column chromatography using an eluent of water and methanol (v/v, 5:1). Finally, MPCDL was successfully got (Yield: 92%).

PCUs based on MDI, PCDL and chain extender were synthesized by two-step solution polymerization method in DMAc under an argon atmosphere. The synthesis route and the corresponding feed ratios are shown in Fig. 1B and Table 1, respectively. Before reaction, MPCDL was dried under vacuum at 100°C for 24 h. After that, PCDL was dried at 100°C under vacuum for 3 h and cooled down for further use. Then, moderate MDI was added into a DMAc solution of PCDL under argon atmosphere at 60°C in a three-neck flask. After that, the reaction mixture was allowed to proceed at 60°C for 1 h to prepare pre-polymer. Then, the chain extender (BDO and MPCDL) was successively added into the flask at 60°C to react for 5 h in the presence of 0.2% stannous octoate. After the termination of reaction, the polymer solution was cooled to ambient temperature and the final product was precipitated in methanol. Finally, the polymer was dried under vacuum for further use.

**Table 1.** The molar ratio of raw materials of PCUs

Samples	MDI (mmol)	PCDL (mmol)	BDO (mmol)	MPCDL (mmol)
PCU40	8	4	4	0
PCU31	8	4	3	1
PCU22	8	4	2	2
PCU13	8	4	1	3
PCU04	8	4	0	4

### Characterization of MPCDL and PCUs

The functional groups of MPCDL and PCUs were first determined *via* the infrared absorption spectra using a FTIR spectrometer (NICOLET 5700, USA). The further structural analysis of MPCDL was obtained *via*^1^H NMR (Bruker AV II-400) and electrospray ionization mass spectrometry (ESI-MS) spectrum. The further structural analysis of PCUs was acquired *via*^1^H NMR and ^31^P NMR. Gel permeation chromatography (GPC) was performed with HLC-8320 (Japan) using N,N-dimethylformamide as eluent. The molecular weights are relative to polymethyl methacrylate standards. The flow rate was 0.4 ml min^−1^ at 40°C. AFM images were acquired using NanoWizard II (Germany). DSC thermograms were obtained using a differential scanning calorimeter (TA, DSC2500) in the range of −70 to 120°C at a heating rate of 10°C min^−1^ under nitrogen.

### Mechanical testing, surface chemical components and hydrophilicity of the PCU films

To detect film-forming performance, mechanical testing of the PCU films was carried out with a universal tensile testing machine (Instron 5567, America) with a 500 mm/min stretching speed. The PCU films were first prepared in a polytetrafluoroethylene mold *via* solvent evaporation method, and the film samples were cut into dumbbell shape with neck width of 5 mm and length of 20 mm. For each sample, the final stress–strain curves were obtained from at least three samples. The surface chemical components of PCU films were analyzed by (XPS(XSAM800, Kratos Ltd, UK) with a monochromatic Al Kα excitation radiation. The containment carbon (C1s = 284.7 eV) and XPSPEAK software were successively used in the calibration of binding energies and the analysis of data. The hydrophilicity of the PCU films was valued by water contact angle *via* Drop Shape Analyzer (DSA100, Krüss). For each sample, same volume of water was dropped on the sample surface and the final data was calculated from at least 15 contact angles.

### Blood compatibility

#### BSA adsorption

The PCUs coatings were prepared on the surface of polished SS sheets *via* solvent evaporation method before the follow-up evaluation. A micro-BCA protein assay kit (Thermo Fisher Scientific, Inc., Waltham, MA) was used to quantify the amounts of BSA absorbed on the different samples surface [47]. The samples were first hydrated in PBS overnight. Then, the same amount of BSA solution (45 mg/ml) was added and incubated at 37°C for 3 h. After that, the samples were then rinsed with PBS to remove the weakly adsorbed BSA. Subsequently, the BSA adsorbed on the surface was eluted with a 2 wt% aqueous solution of SDS. Finally, the concentrations of BSA in the solutions of each set of samples were calculated from at least six sheets.

#### Fibrinogen denaturation and adsorption tests

Enzyme-linked immunosorbent assay was used in the tests of fibrinogen denaturation and adhesion of different samples [48, 49]. First, 3 mg/ml of human fibrinogen solution and 42 mg/ml of BSA solution was obtained *via* PBS. Then, the same volume of fibrinogen solution was added onto every sample surface and incubated at 37°C for 1 h. After that, the weakly adsorbed fibrinogen was removed with PBS [50]. Furthermore, the samples were incubated with 1 wt% BSA solution to blocked the fibrinogen, after which the samples were washed with PBS and incubated with rabbit anti-human fibrinogen gamma chain antibody (1/500 dilution in PBS, product No: bs-1240G, Bioss) at 37°C for 1 h. After that, the samples were incubated with goat anti-rabbit IgG/HRP antibody (1/1000 dilution in PBS, product No.: bs-0295G-HRP, Bioss) at 37°C for 1 h. Subsequently, 3,3,5,5-tetramethylbenzidine solution (TMB) was used to react with HRP. After 8 min, chromogenic reaction between the HRP and TMB was stopped *via* sulfuric acid. Then, the results of fibrinogen denaturation were got *via* the detection of reaction solution at 450 nm using microplate reader. Besides, another set of samples was incubated with goat anti-human fibrinogen/HRP-conjugated antibody (1/100 dilution in PBS, product No: bs-1240G-HRP, Bioss) at 37°C for 1 h to get the results of fibrinogen adsorption. Similarly, the results of fibrinogen adsorption were got at 450 nm using microplate reader after the reaction of TMB and HRP.

#### Platelet adhesion and whole-blood test

Before test, the platelet rich plasma (PRP) was obtained by the centrifugation of fresh human whole-blood at l500 rpm. Immediately, the same volume of PRP was added onto each surface of samples and incubated at 37°C for 1 h. Furthermore, the samples were rinsed with PBS three times to remove non-firmly adsorbent platelets. After that, the samples were fixated with 2.5 wt% glutaraldehyde solution overnight. For the whole-blood test [51], the same volume (0.5 ml) of fresh human whole-blood was added onto each well of a 24-well plate containing samples. After 30 min incubation at 37°C, the samples were cleaned with PBS and fixed by 2.5% glutaraldehyde overnight. After fixation, all the samples were respectively dehydrated, dealcoholized, critical point drying and gold spraying. At last, all the samples were observed *via* SEM.

### *In vitro* ECs and SMCs culture

M199 culture media (Hyclone, USA) with 20 μg/ml EC growth supplement (Millipore, Inc.) and 10% fetal bovine serum (FBS) was used in the ECs culture. Modified Eagle’s medium/F12 media with 15% FBS supplement was used in the SMCs culture. The ECs and SMCs were seeded onto the samples surface with a density of 1.0 × 10^4^ cells/sample and 3 × 10^4^ cells/sample, respectively. Cell culture was performed in an incubator containing 5% CO_2_ at 37°C for 4, 24 and 72 h. After the scheduled time (4, 24 and 72 h), all the cells on the samples surface were stained with Calcein-AM (Cal-AM, AAT Bioquest) for 20 min and then fixed with glutaraldehyde (2.5%) for 12 h. After that, all the samples were cleaned with physiological saline three times. Thereafter, fluorescence images of all samples were got *via* an Olympus fluorescent microscope (IX51, Japan). Besides, the cellular metabolic activities after 24 and 72 h were studied *via* the cell counting kit-8 (CCK-8, Dojindo) assay. After 24 or 72 h culture, the culture solution of each sample was replaced with 350 μl of fresh culture solution containing CCK-8 reagent and then incubated for another 3 h. The same volume of the culture solution was taken from each sample and added into a 96-well plate. Finally, a microplate reader was used to measure the absorbance of final culture solution at 450 nm.

### Animal experiments

#### *Ex vivo* blood perfusion experiment

The dynamic evaluation of blood perfusion experiment [52–54] was conducted using adult New Zealand white rabbits weighing of ∼3 kg each. The schematic diagram was shown in Fig. 8A. First, the polymer films formation was prepared on the surface of SS foil (∼8 mm × 10 mm) by solvent evaporation method. Then, the samples were placed into the heparinized sterile polyvinyl chloride (PVC) catheters. After that, surgical indwelling needles were used connected with carotid artery and jugular vein. Immediately, the PVC catheters containing samples were connected with the surgical indwelling needles and allowed the blood to flow in the circulation path. After 1 h of dynamic blood circulation, the catheters around the samples were cut off. Then, the cross section of catheter containing sample and the blood clot on the sample surface were recorded *via* digital pictures. After that, occlusion ratio was calculated by measuring the cross-section diameter of the circulating tube. Then, the samples were immediately fixed with 2.5 wt% glutaraldehyde solution overnight, dehydrated and dealcoholized in turns. In addition, the thrombus weight was calculated *via* the weight difference before and after *ex vivo* blood perfusion experiment. At last, microscopic state of thrombus on the samples was observed *via* SEM.

#### *In vivo* tissue response evaluation

Healthy rats (Sprague Dawley, SD) were used for the evaluation of the tissue response. SS sheets and different PCUs-coated sheets were subcutaneously implanted in different location of the back of the rats [53]. After 3 and 9 weeks, the samples with local surrounding tissues were first collected. Then, paraformaldehyde was used for the fixation of the collected tissues at 25°C for 72 h. After that, the tissues were then rinsed with PBS after fixation and then followed dehydration *via* graded ethanol, saturation in xylene and paraffin-embedded. Then, the paraffin samples were cut into slices and then carried out hematoxylin and eosin (H&E) staining. At last, fibrous capsules around different samples were observed and recorded *via* microscope.

## Results and discussion

### Characterization of MPCDL and PCUs

Structure analysis of MPCDL *via* FTIR, ^1^H NMR and ESI-MS are shown in Supplementary Figs S1–S4. As shown in Supplementary Fig. S1, the peaks at 968, 1479 and 1724 cm^−1^ were respectively assigned to the structure of –N^+^(CH_3_)_3_, –P = O and –C = O. The specific group (–SH) ascribes to the MP molecule was disappeared at 2556 cm^−1^. Besides, the ^1^H NMR results (Supplementary Fig. S2) further proved the structure of MP. The peak at 4.01–4.11, 3.80 and 3.51 ppm was corresponded to the structure of –OC*H*_2_C*H*_2_OPOC*H*_2_C*H*_2_N– and –C*H*–OH. The peaks at 5.18 ppm and 4.98 ppm were ascribed to the hydroxyl. The peaks at 3.29 and 3.10 ppm were corresponded to the structure of –C*H*_2_OH and –N^+^(C*H*_3_)_3_. The peaks at 2.39–2.76 and 1.09 ppm were associated with the protons of –C*H*_2_SC*H*_2_C*H*C*H*_3_. The result of ESI-MS was corresponded to the theoretical molecular weight (M + H, Supplementary Fig. S4). In summary, these results strongly proved the successful synthesis of MPCDL.

**Figure 2.**
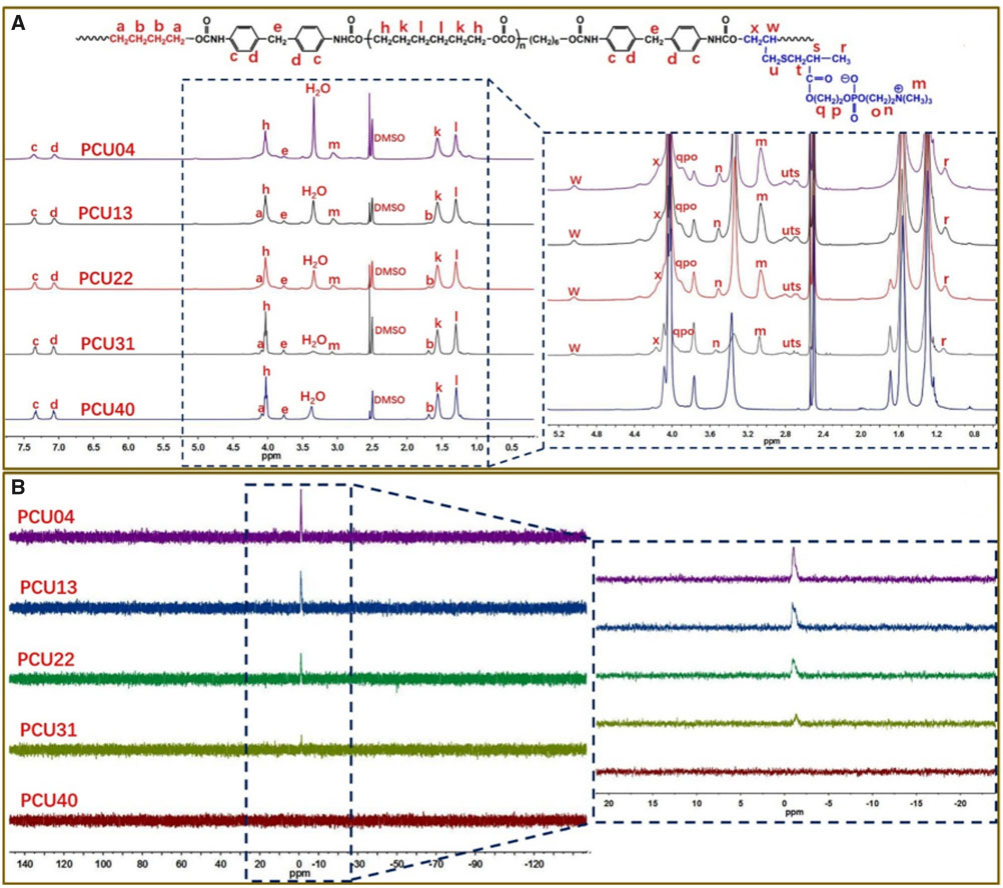
(**A**) The spectra of ^1^H NMR of PCU40, PCU31, PCU22, PCU13 and PCU04. (**B**) The spectra of ^31^P NMR of PCU40, PCU31, PCU22, PCU13 and PCU04

The structure of PCUs was first analyzed *via* FTIR (Supplementary Fig. S5). The peaks at 2939 and 2861 cm^−1^ were assigned to the methylene groups. The peaks at 1743 and 1599 cm^−1^ were respectively corresponded to the carbonyl groups and aromatic ring. Particularly, the peak of –NH belongs to amino acid ester groups was appeared at 1536 cm^−1^ and the peak of –N = C=O was not appeared at the scope of 2240–2280 cm^−1^. Then, the structure of PCUs was studied in detail *via*^1^H NMR and ^31^P NMR. As shown in Fig. 2A, the chemical shift values at 4.03, 1.57 and 1.30 ppm were ascribed to the methylene groups of PCDL segment. The peaks at 7.35, 7.06 and 3.77 ppm were assigned to the protons of MDI segment. Moreover, the proton peaks belong to BDO segment and MPCDL segment displayed regular change due to the difference of formulation. The peaks at 4.09 and 1.69 ppm were ascribed to the methylene groups of BDO segment. As shown in the Fig. 2A, the chemical shift values of MPCDL segment were also displayed in the local amplification figure of ^1^H NMR. The ^1^H NMR spectra provides a more clearly understanding of the structure of PCUs. As shown in Fig. 2B, the chemical shift values around −1.0 ppm was ascribed to the structure of –P = O. Moreover, the peaks of ^31^P NMR were almost corresponded to the formulation and molecular design of PCUs. In summary, the results of FTIR and NMR were strongly proved the successful synthesis of PCUs.

### Surface chemical components of the PCU films

**Figure 3.**
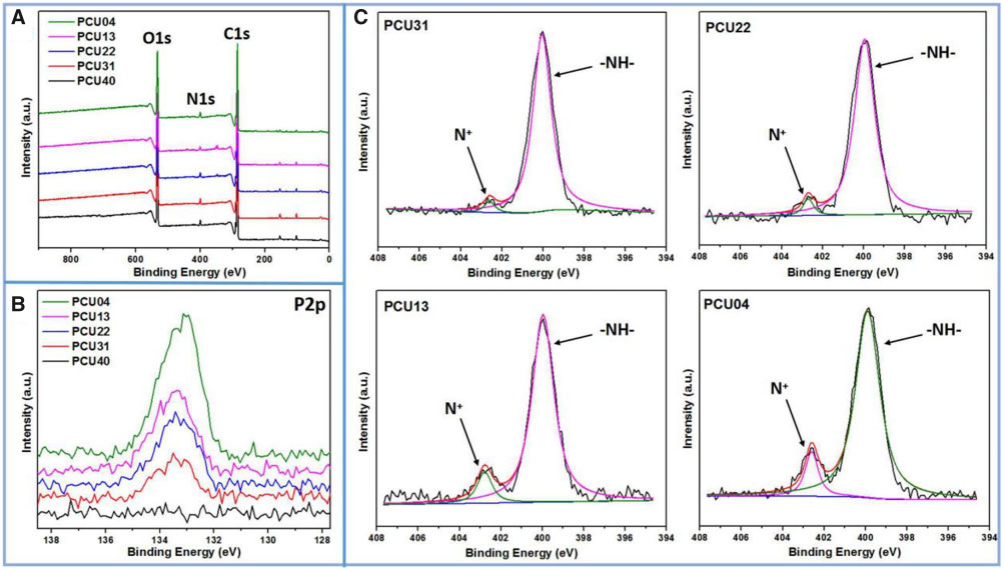
(**A**) XPS full spectrum of PCUs; (**B**) results of high-resolution P2p of PCUs; (**C**) curve-fitting results of high-resolution N1s of PCUs

Surface chemical components of biomaterials would produce a certain influence on biocompatibility, such as protein adsorption and cell proliferation. Herein, to understand the content change of surface element and structure, XPS was carried out. From the results of XPS whole spectrum shown in Fig. 3A, main elements including nitrogen (N), oxygen (O) and carbon (C) were observed. To better understand the difference of surface chemical components, the high-resolution XPS was also carried out and the results were shown in Fig. 3B and C. The peak at 133.4 eV represents the binding energy of P2p, which can be ascribed to the –OPOCH_2_– of phosphorylcholine group. It could be observed that the content of P increases as the amount of MPCDL increases, indicating the enrichment of phosphorylcholine groups on the surface. Besides, curve-fitting results of high-resolution N1s were shown in Fig. 3C. Similarly, obvious increase in the contents of N^+^ could be found as the amount of MPCDL increases. Moreover, the elemental composition and ratios were shown in Table 2. The ratios of N^+^/N and P/C increase as the amount of MPCDL increases, which consistent with the results shown in Fig. 3B and C. In summary, the XPS results provide a better understanding on the surface components of PCU films.

**Table 2.** The elemental composition and ratios of PCUs

Samples	C (%)	N (%)	O (%)	P (%)	P/C (%)	N+/N (%)
PCU40	74.5	1.9	23.5	0	0	0
PCU31	74.7	2.5	22.7	0.1	0.0013	3.5
PCU22	76.2	2.0	21.6	0.2	0.0026	4.7
PCU13	79.0	1.6	19.2	0.3	0.0038	9.9
PCU04	76.0	2.5	21.1	0.4	0.0053	11.4

### Characterizations of the PCUs

**Figure 4.**
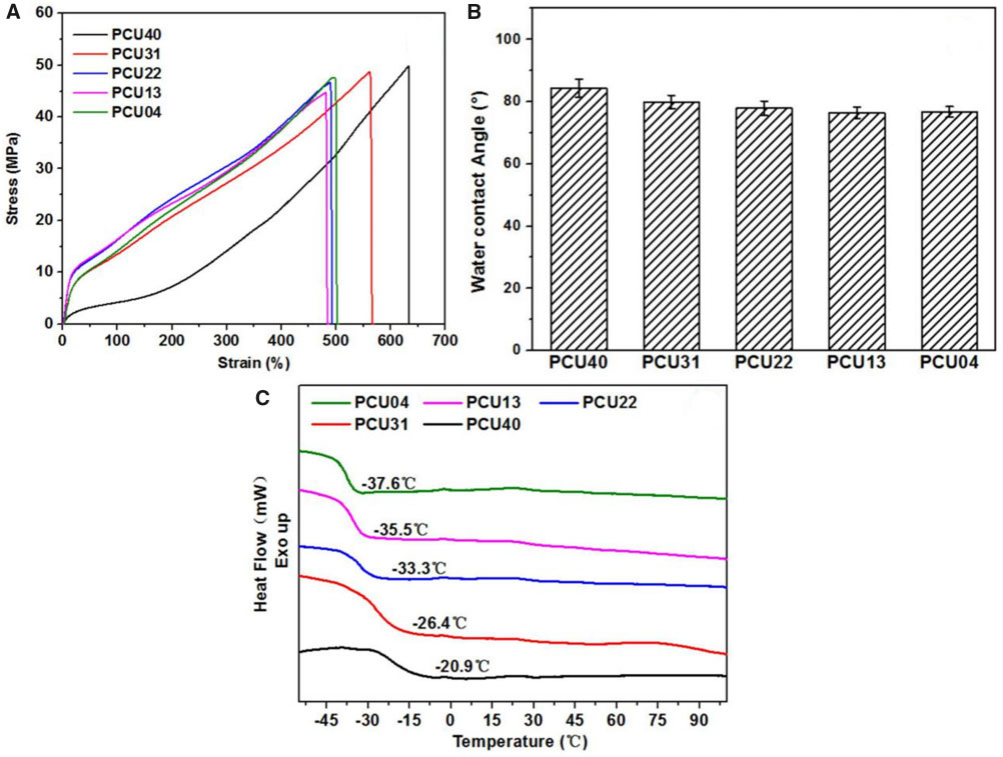
(**A**) Stress–strain curves of PCUs. (**B**) WCA results after dropping of water on the surface of the PCUs films. (**C**) DSC curves of PCUs, the heating rate of 10°C/min

The physical and chemical properties of PCUs film were successively studied *via* uniaxial tensile, WCA and DSC test. The stress–strain curves of different PCUs sample were shown in Fig. 4A and the data were listed in Table 3. All the PCUs films exhibited excellent tensile properties with strain at break of 484–633% and ultimate stress of 44.6–49.7 MPa. Besides, the modulus (38–73 MPa) of PCUs containing MPCDL is much higher than that of PCU40. According to related studies [55], the polarity difference between the hard and the soft domains may be enhanced with the increase in phosphorylcholine groups. Therefore, the higher microphase separation degree of PCUs containing MPCDL will lead to higher modulus compared with PCU40. In general, the physical properties of the biomedical polymers are essential for the performance requirements of medical devices. The mechanical properties of proposed PCUs films meet most clinical requirements of blood-contacting implants such as intravenous catheter. The surface wettability of samples may cause effects on the adhesion performance. For this reason, hydrophilicity of different PCUs films was measured with WCA. As shown in Fig. 4B, only slight reduction of WCA of PCU22, PCU13 and PCU04 samples was observed. Thus, the increase in MPCDL content virtual did not affect the hydrophilicity i.e. the aggregation of phosphocholine is rather weak. However, according to the DSC curves of PCUs (Fig. 4C), obvious changes were observed in the glass transition temperature (*T*_g_) of soft segment [56–58] of different PCUs samples. This phenomenon indicated that the degree of microphase separation of PCUs will increased with the increase in MPCDL content, which consists with the results of AFM (see Supplementary Fig. S6). In general, these physicochemical properties of PCUs film may affect the performance of following biological response.

**Table 3.** GPC and strain–stress results of PCUs

Samples	*M*_n_ (10^4^)	*M*_w_ (10^4^)	*M*_w_/*M*_n_	Ultimate stress (MPa)	Tensile modulus (MPa)	Strain at break (%)
PCU40	3.8	8.8	2.3	49.7	13.1	632.5
PCU31	3.0	8.0	2.7	48.6	38.8	561.5
PCU22	1.7	7.0	4.1	46.4	70.6	489.1
PCU13	1.3	4.6	3.5	44.6	72.6	483.9
PCU04	2.1	6.3	3.0	47.4	43.3	497.2

### Adhesion tests of BSA and fibrinogen

**Figure 5.**
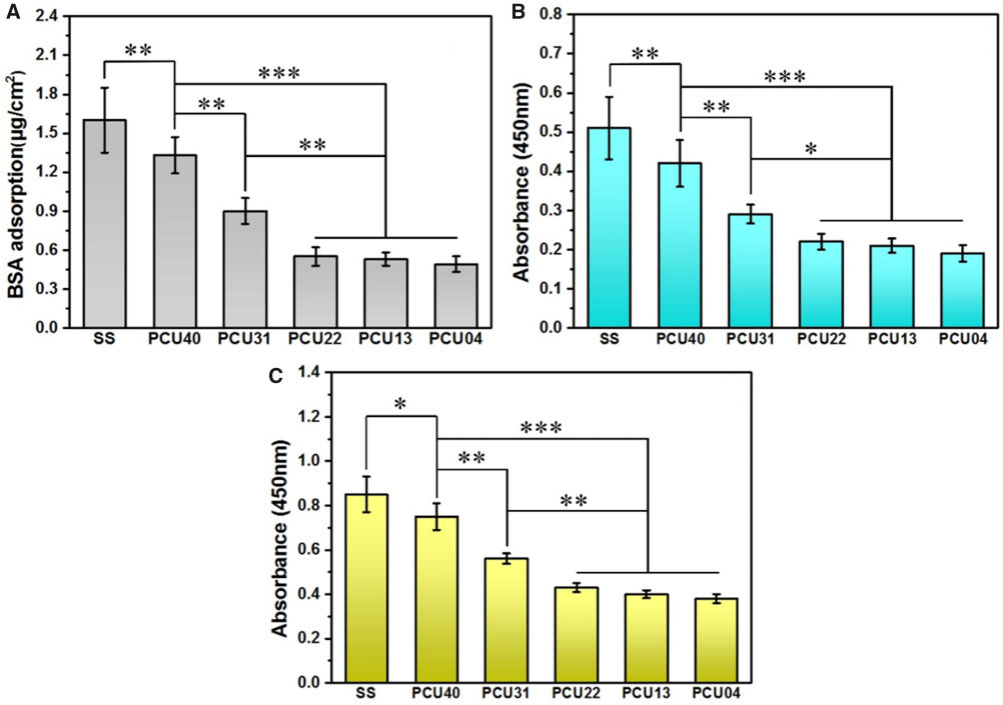
(**A**) BSA adsorption, (**B**) fibrinogen adsorption and (**C**) fibrinogen denaturation results on the surface of SS, PCU40, PCU31, PCU22, PCU13 and PCU04 (*P < 0.05, **P < 0.01 ***P < 0.001, mean ± SD, *N* = 5)

As the most abundant protein in the blood, albumin is always associated with the process of coagulation and other host response. To determine the adhesion conditions of albumin on different PCUs surface, BSA was used in the test *via* micro-BCA protein assay kit. As shown in Fig. 5A, the most amount of adsorbed BSA was observed on SS, while a marked decrease was observed on PCU40. With the increasing in MPCDL content in PCUs (in the order of PCU40 > 31 > 22 > 13 > 04), BSA adsorption keeps decreasing. Similar phenomenon can be also observed in fibrinogen adhesion and denaturation tests (Fig. 5B and C). These results strongly indicate that more MPCDL content in PCUs owns stronger ability in resisting protein adsorption and denaturation. This behavior is usually associated with the synergy of microphase separation and aggregation of phosphocholine.

### Platelet adhesion and whole-blood test

**Figure 6.**
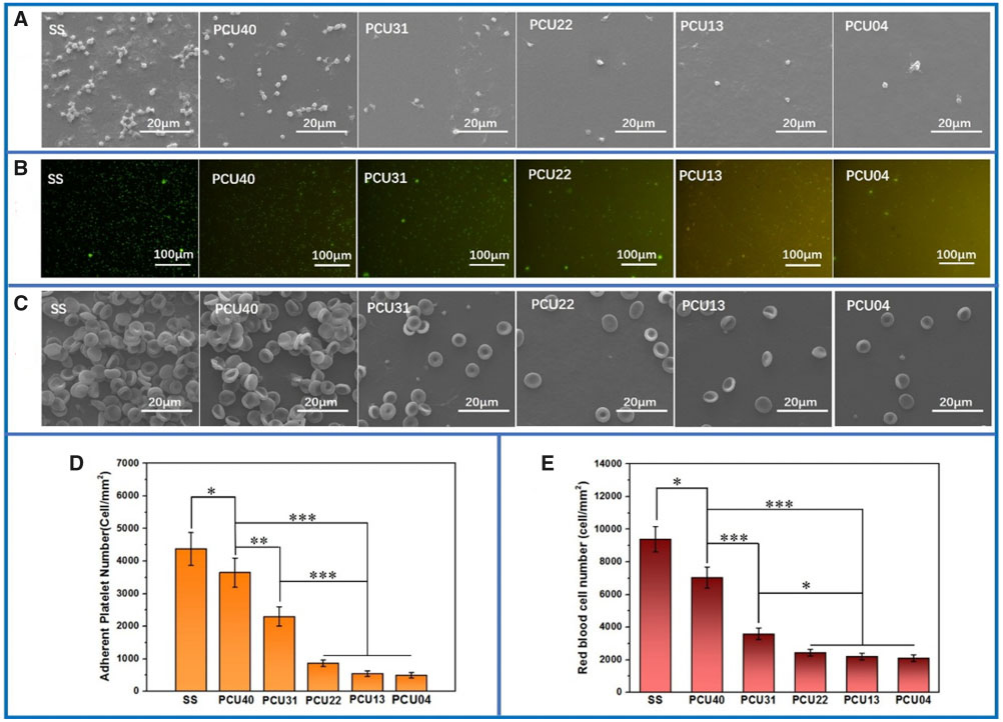
(**A**) SEM results and (**B**) rhodamine staining of platelets adhered on different samples surface. (C) SEM results of whole-blood adhered on different samples surface. (**D**) Quantitative analysis of platelets and (**E**) red blood cells adhered on different samples surface (*P < 0.05, **P < 0.01 ***P < 0.001, mean ± SD, *N* = 5)

As one of the most important components in the blood, platelets are often used to value the hemocompatibility of biomaterials. Here, the adhesion behavior of platelets on different surfaces was observed with SEM (Fig. 6A). Obviously, the SS samples showed the poor platelets compatibility with large number of platelets adhesion and aggregation. After the preparation of PUs on the substrate surface, the status of platelet aggregation and activation was observed with a certain improvement. With the further increasing in MPCDL content in PCUs, less platelets were observed, indicating strong ability of inhibiting platelet adhesion and aggregation. The rhodamine staining results (Fig. 6B) consistent with the SEM pictures, where the platelet numbers on high MPCDL content surfaces (PCU22, PCU13 and PCU04) were significantly lesser than that on the others (SS, PCU40 and PCU31). Similar phenomenon can be also observed in the whole-blood test (Fig. 6C). Finally, the statistics analysis directly showed a quantitative result, which confirms that the count results of adherent platelet (Fig. 6D) and red blood cell (Fig. 6E) numbers decreases with the increasing in MPCDL content. Therefore, we can draw a conclusion that the samples containing high content of MPCDL has better blood compatibility and may be useful in the surface modification of the medical metals.

### ECs culture

**Figure 7.**
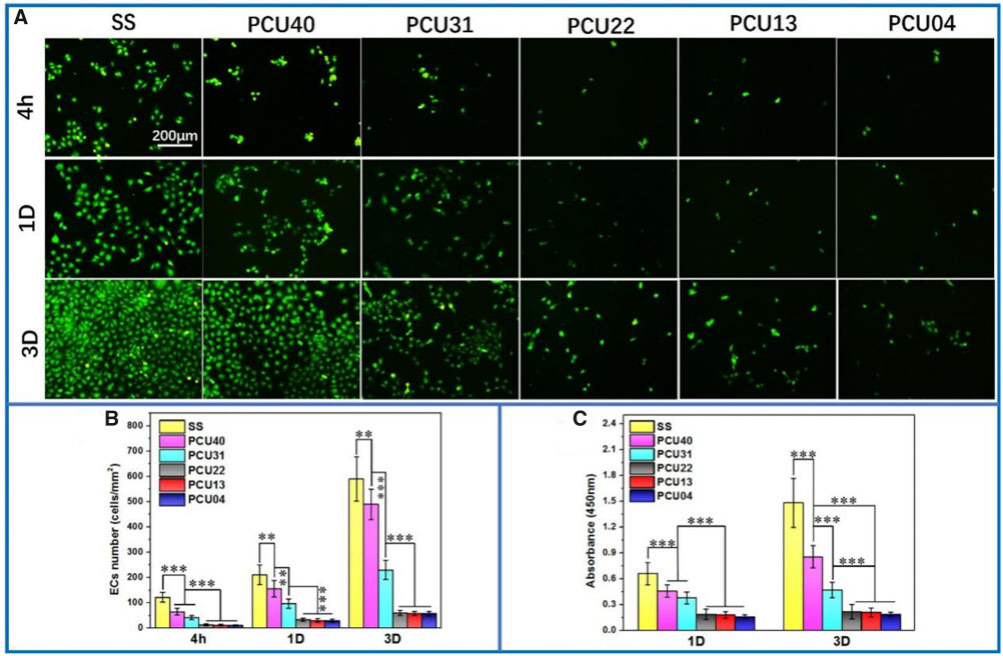
(**A**) Cal-AM staining of ECs on the surface of SS, PCU40, PCU31, PCU22, PCU13 and PCU04. (**B**) The count analysis of ECs at 4 h, 1 and 3 days. (**C**) The results of CCK-8 of ECs of SS, PCU40, PCU31, PCU22, PCU13 and PCU04 (**P < 0.01, ***P < 0.001, mean ± SD, *N* = 5)

Cell culture as another important method was used in evaluation of cell compatibility and anti-fouling ability of implant devices. As shown in Fig. 7A, SS showed a marked larger number of adherent ECs and smaller cytoskeleton than PCUs after 4 h culture. This tendency keeps unchanged with the increase in culture time to 1 and 3 days. Notably, when the chain extender content was more than 50% (i.e. PCU22, PCU13 and PCU04), the ECs culture displayed the least adherent amount and the smallest cytoskeleton. The detailed deviations were then proved by quantitative count analysis (Fig. 7B), consisted well with Cal-AM staining pictures. Besides, the cell viability of ECs was determined *via* CCK-8 assay since the absorbance value of CCK-8 solution is positive correlation with the cell vitality (Fig. 7C). The cytoskeleton of ECs on PCUs samples was also consistent with the results of CCK-8. All these ECs culture results indicating the weak adhesive ability of ECs.

### SMCs culture

**Figure 8.**
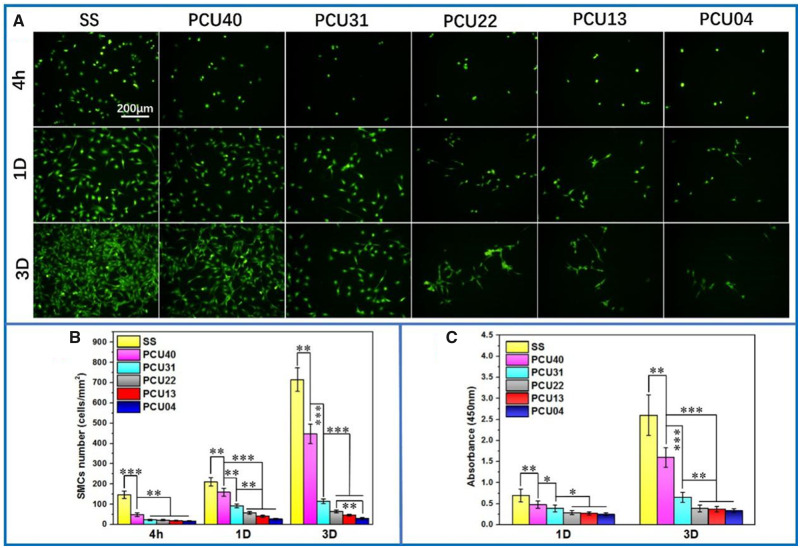
(**A**) Cal-AM staining of SMCs on the various samples surface at 4 h, 1 and 3 days. (**B**) The count analysis and (**C**) the results of CCK-8 of SMCs on SS, PCU40, PCU31, PCU22, PCU13 and PCU04 (*P < 0.05, **P < 0.01, ***P < 0.001, mean ± SD, *N* = 5)

The proliferation of SMCs is related to intimal hyperplasia on the surface of intravascular implant devices, which is an important indicator in evaluation of anti-fouling properties of biological materials. Therefore, SMCs were seeded and cultured on different samples and the results were shown in Fig. 8. As control sample, the SS showed the largest number of cell adhesion after 4 h culture. Whereas, only small number of SMCs were found in the fluorescent pictures of PCUs (Fig. 8A). After 1 and 3 days culture, the number of SMCs on PCUs was significantly lesser than that of SS samples. Particularly, the cytoskeleton of SMCs on the PCU22, PCU13 and PCU04 samples was smallest, indicating the weakest adhesive ability and cell viability. Similar to ECs, the detailed deviations of SMCs on different surfaces were also proved by quantitative count analysis (Fig. 8B), where similar tendency was observed. Besides, the cell viability of ECs was determined *via* CCK-8 (Fig. 8C), which were also consistent with the results of Cal-AM staining. On the whole, the phospholipid-based PCUs could effectively inhibit SMCs hyperplasia and may be useful in the applications of intravascular implants.

### *Ex vivo* blood circulation

**Figure 9.**
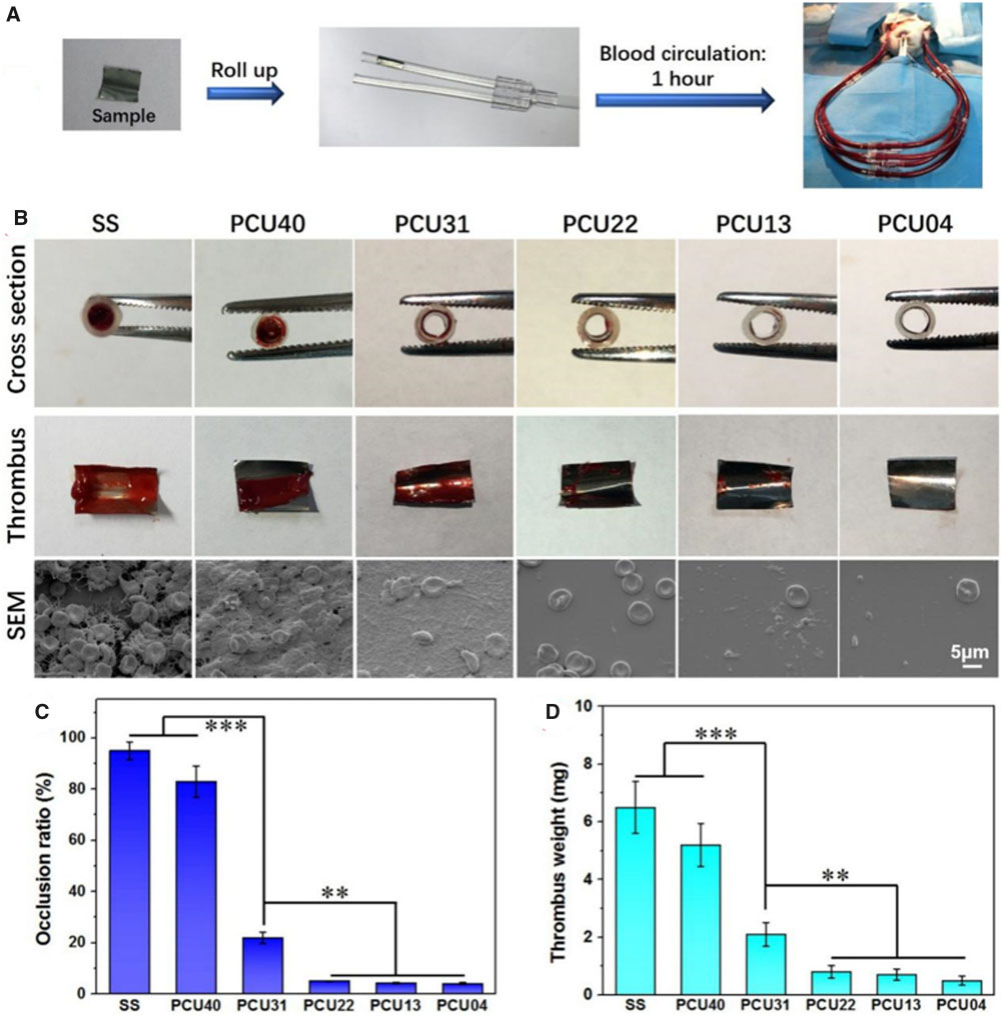
*Ex vivo* evaluation of dynamic blood on various samples. (**A**) The assembly process of *ex vivo* blood circulation. (**B**) The results of cross section around samples, pictures of thrombus on samples and SEM photos of the thrombus on samples. (**C**) The results of occlusion ratio by measuring the cross-section diameter of the circulating catheter. (**D**) The results of thrombus weight on different samples surface (**P < 0.01, ***P < 0.001, mean ± SD, *N* = 5)

Blood-contacting devices usually facing with the host responses of the organization such as acute blood coagulation. For this reason, it is necessary to reduce acute blood clotting response. *Ex vivo* evaluation of dynamic blood on various samples was carried out (Fig. 9). As shown in Fig. 9B, digital photos of cross section around samples displayed the difference of occlusion of various samples. Moreover, digital photos of thrombus on samples intuitively demonstrated the conditions of acute blood coagulation. Severe occlusion and thrombus were observed on the SS and PCU40, whereas no detectable occlusion and slight thrombosis were observed in the PCUs containing MPCDL. Particularly, the thrombosis on the samples of PCU22, PCU13 and PCU04 was the slightest, indicating the superior ability of inhibiting thrombus. SEM results (Fig. 9B) also confirmed that the PCU22, PCU13 and PCU04 could impressively suppressed thrombus including platelet activation, fibrin formation and red blood cell adhesion. Statistical analysis of occlusion ratio (Fig. 9C) of the cross-section reveals a remarkable reduction in the phospholipid-based PCUs-coated 316L SS circuit. Besides, statistical analysis of weight (Fig. 9D) of the thrombus was also significantly reduced in the phospholipid-based PCUs-coated samples. Overall, *ex vivo* blood circulation strongly proved the possibility of phospholipid-based PCUs for the blood-contacting implants.

### *In vivo* tissue response

**Figure 10.**
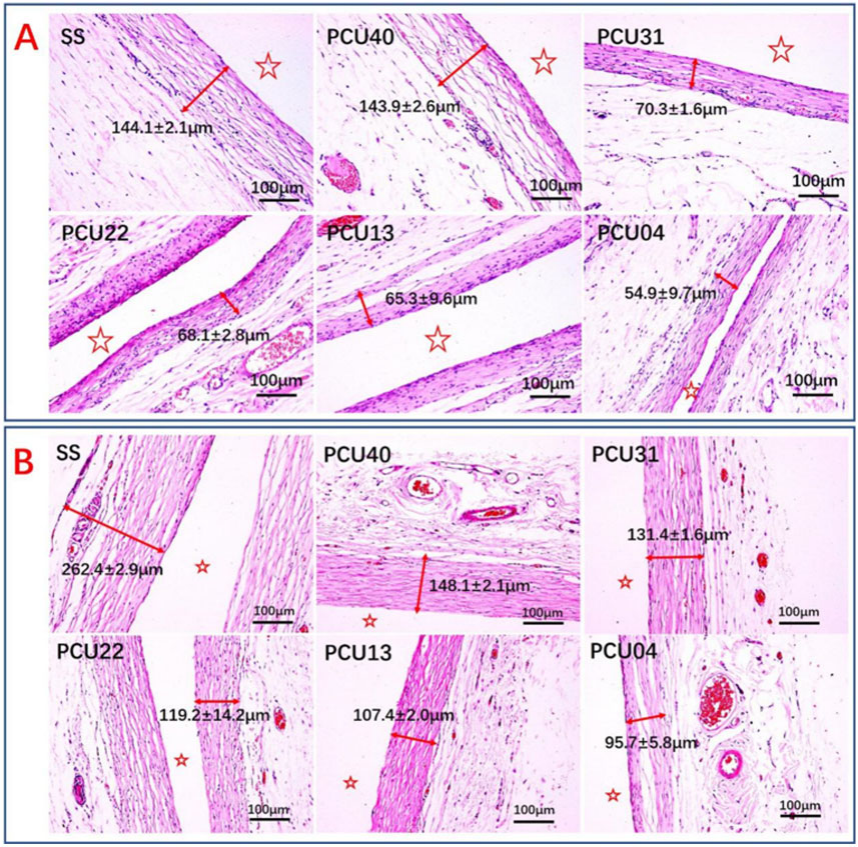
H&E staining and thickness results of the local tissues around different samples after (**A**) 3 and (**B**) 9 weeks of subcutaneous implantation. The sample-implanted location is marked by ⋆

Tissue response, such as inflammatory response, is also a crucial factor for the research of novel implant devices. Subcutaneous implantation in the back of SD rats was carried out to determine tissue response property of PCUs. After implantation for 3 and 9 weeks, different thickness of the fibrous capsule can be observed on the samples. As shown in Fig. 10A, the fibrous capsule of SS and PCU40 showed the greater thickness, indicting serious inflammatory response. However, PCUs containing MPCDL tended to prevent the occurrence of inflammatory response with thin fibrous capsule. Moreover, samples containing high content of MPCDL own gentle tissue response with thinner capsule. As the extension of implanted time, the fibrous capsule will become mature and thicker. H&E staining results after 9 weeks were displayed in Fig. 10B, thinner capsule on the PCU22, PCU13 and PCU04 samples than those on SS and PCU40 and PCU31 were also observed. Both short- and long-term tissue responses all demonstrated that the PCU22, PCU13 and PCU04 could effectively prevent the occurrence of tissue response.

## Conclusions

In this study, to decrease non-specific adhesion and inflammatory response, a phospholipid-based PCUs is developed *via* two-step solution polymerization. The results of stress–strain curves exhibited outstanding tensile properties of PCUs films. Besides, the microphase separation degree could be adjusted *via* the differences of structure hence leading to different biological responses. After the surface modification of SS sheets with PCUs. A series experimental result including platelet adhesion, BSA adsorption, fibrinogen adsorption and whole-blood test indicated a strong ability in reducing non-specific adhesion and thrombosis. ECs and SMCs culture further revealed good properties of anti-cell adhesion. Finally, animal experiments including *ex vivo* perfusion experiment and subcutaneous inflammation test indicated the remarkable abilities of anti-thrombosis and histocompatibility. These investigations high light the strong anti-adhesion property of phospholipid-based PCUs, which may be applied to the blood-contacting devices such as intravenous catheter or antithrombotic surface in the future.

## Funding

This work was financially supported by the National Key Research and Development Program of China (2016YFC1100402), the International Cooperation Project by Science and Technology Department of Sichuan Province (2020YFH0103), the National Natural Science Foundation of China (NSFC Project 81801853), Postdoctoral Science Foundation of China (2018M633400) and Sichuan Science and Technology Program (19GJHZ0058). 

Conflict of interest 

The authors declare that they have no known competing financial interests or personal relationships that could have appeared to influence the work reported in this paper.

## Supplementary Material

rbaa037_Supplementary_Data

## References

[1] Golabchi A, Wu B, Cao B et al Zwitterionic polymer/polydopamine coating reduce acute inflammatory tissue responses to neural implants. Biomaterials 2019;225:119519. 31600673 10.1016/j.biomaterials.2019.119519 PMC6896321

[2] Zhang W, Hu J, Zhou Y et al Latex and a ZnO-based multi-functional material for cardiac implant-related inflammation. Biomater Sci 2019;7:4186–94. 31384848 10.1039/c9bm00952c

[3] Harbers GM, Emoto K, Greef C et al Functionalized poly (ethylene glycol)-based bioassay surface chemistry that facilitates bio-immobilization and inhibits nonspecific protein, bacterial, and mammalian cell adhesion. Chem Mater 2007;19:4405–14. 18815622 10.1021/cm070509u PMC2546567

[4] Zhang H, Chiao M. Anti-fouling coatings of poly (dimethylsiloxane) devices for biological and biomedical applications. J Med Biol Eng 2015;35:143–55. 25960703 10.1007/s40846-015-0029-4 PMC4414934

[5] Mariani E, Lisignoli G, Borzì RM et al Biomaterials: foreign bodies or tuners for the immune response? IJMS 2019;20:636. 10.3390/ijms20030636 PMC6386828 30717232

[6] Xu X, Wang L, Luo Z et al Facile and versatile strategy for construction of anti-inflammatory and antibacterial surfaces with polydopamine-mediated liposomes releasing dexamethasone and minocycline for potential implant applications. ACS Appl Mater Interfaces 2017;9:43300–14. 29140074 10.1021/acsami.7b06295

[7] Singh DP, Herrera CE, Singh B et al Graphene oxide: an efficient material and recent approach for biotechnological and biomedical applications. Mater Sci Eng C 2018;86:173–97. 10.1016/j.msec.2018.01.004 29525091

[8] Qiu J, Hameau A, Shi X et al Fluorescent phosphorus dendrimers: towards material and biological applications. ChemPlusChem 2019;84:1070–80. 31943953 10.1002/cplu.201900337

[9] Zhan R, Pan Y, Manghnani PN et al AIE polymers: synthesis, properties, and biological applications. Macromol Biosci 2017;17:1600433. 10.1002/mabi.201600433 27996201

[10] Cai H, Huang Y-L, Li D. Biological metal–organic frameworks: structures, host–guest chemistry and bio-applications. Coord Chem Rev 2019;378:207–21.

[11] Akindoyo JO, Beg M, Ghazali S et al Polyurethane types, synthesis and applications—a review. RSC Adv 2016;6:114453–82.

[12] Guelcher SA. Biodegradable polyurethanes: synthesis and applications in regenerative medicine. Tissue Eng B 2008;14:3–17. 10.1089/teb.2007.0133 18454631

[13] Xu C, Yepez G, Wei Z et al Synthesis and characterization of conductive, biodegradable, elastomeric polyurethanes for biomedical applications. J Biomed Mater Res A 2016;104:2305–14. 27124702 10.1002/jbm.a.35765 PMC10947274

[14] Marzec M, Kucińska-Lipka J, Kalaszczyńska I et al Development of polyurethanes for bone repair. Mater Sci Eng C 2017;80:736–47. 10.1016/j.msec.2017.07.047 28866223

[15] Cheng X, Fei J, Kondyurin A et al Enhanced biocompatibility of polyurethane-type shape memory polymers modified by plasma immersion ion implantation treatment and collagen coating: an *in vivo* study. Mater Sci Eng C 2019;99:863–74. 10.1016/j.msec.2019.02.032 30889761

[16] Zhao X, Dong R, Guo B et al Dopamine-incorporated dual bioactive electroactive shape memory polyurethane elastomers with physiological shape recovery temperature, high stretchability, and enhanced C2C12 myogenic differentiation. ACS Appl Mater Interfaces 2017;9:29595–611. 28812353 10.1021/acsami.7b10583

[17] Hao H, Shao J, Deng Y et al Synthesis and characterization of biodegradable lysine-based waterborne polyurethane for soft tissue engineering applications. Biomater Sci 2016;4:1682–90. 27709130 10.1039/c6bm00588h

[18] Wang B, Ye Z, Tang Y et al Fabrication of nonfouling, bactericidal, and bacteria corpse release multifunctional surface through surface-initiated RAFT polymerization. IJN 2016;12:111–25. 28053527 10.2147/IJN.S107472 PMC5191580

[19] Xu B, Liu Y, Sun X et al Semifluorinated synergistic nonfouling/fouling-release surface. ACS Appl Mater Interfaces 2017;9:16517–23. 28417636 10.1021/acsami.7b03258

[20] Wu F, Li J, Zhang K et al Multifunctional coating based on hyaluronic acid and dopamine conjugate for potential application on surface modification of cardiovascular implanted devices. ACS Appl Mater Interfaces 2016;8:109–21. 26654689 10.1021/acsami.5b07427

[21] Li J, Zou D, Zhang K et al Strong multi-functions based on conjugating chondroitin sulfate onto an amine-rich surface will direct the vascular cell fate for cardiovascular implanted devices. J Mater Chem B 2017;5:8299–313. 32264499 10.1039/c7tb02162c

[22] Chen S, Luo C, Wen W et al Fabrication, antibacterial activity and cytocompatibility of quaternary ammonium chitooligosaccharide functionalized polyurethane membrane *via* polydopamine adhesive layer. Mater Sci Eng C 2018;93:319–31. 10.1016/j.msec.2018.08.006 30274064

[23] Wang R, Xiang T, Zhao W-F et al A facile approach toward multi-functional polyurethane/polyethersulfone composite membranes for versatile applications. Mater Sci Eng C 2016;59:556–64. 10.1016/j.msec.2015.10.058 26652408

[24] Kwon HJ, Lee Y, Seon GM et al Zwitterionic sulfobetaine polymer-immobilized surface by simple tyrosinase-mediated grafting for enhanced antifouling property. Acta Biomater 2017;61:169–79. 28782724 10.1016/j.actbio.2017.08.007

[25] Liu Z, Fang L, Delaittre G et al Heparinized polyurethane surface *via* a one-step photografting method. Molecules 2019;24:758. 10.3390/molecules24040758 PMC6412568 30791534

[26] Qiu X, Lee BL-P, Ning X et al End-point immobilization of heparin on plasma-treated surface of electrospun polycarbonate-urethane vascular graft. Acta Biomater 2017;51:138–47. 28069505 10.1016/j.actbio.2017.01.012 PMC5346065

[27] Fang J, Zhang J, Du J et al Orthogonally functionalizable polyurethane with subsequent modification with heparin and endothelium-inducing peptide aiming for vascular reconstruction. ACS Appl Mater Interfaces 2016;8:14442–52. 27224957 10.1021/acsami.6b04289

[28] Choi WS, Joung YK, Lee Y et al Enhanced patency and endothelialization of small-caliber vascular grafts fabricated by coimmobilization of heparin and cell-adhesive peptides. ACS Appl Mater Interfaces 2016;8:4336–46. 26824876 10.1021/acsami.5b12052

[29] Jia R-P, Zong A-X, He X-Y et al Synthesis of newly fluorinated thermoplastic polyurethane elastomers and their blood compatibility. Fibers Polym 2015;16:231–8.

[30] Chan-Chan L, Tkaczyk C, Vargas-Coronado R et al Characterization and biocompatibility studies of new degradable poly (urea) urethanes prepared with arginine, glycine or aspartic acid as chain extenders. J Mater Sci: Mater Med 2013;24:1733–44. 23615787 10.1007/s10856-013-4931-4

[31] Castillo-Cruz O, Aviles F, Vargas-Coronado R et al Mechanical properties of l-lysine based segmented polyurethane vascular grafts and their shape memory potential. Mater Sci Eng C 2019;102:887–95. 10.1016/j.msec.2019.04.073 31147060

[32] Aydin A, Demirci F, Orhan M et al Preparation of breathable polyurethane membranes with quaternary ammonium salt diols providing durable antibacterial property. J Appl Polym Sci 2019;136:47133.

[33] Speckhard TA, Hwang KK, Yang CZ et al Properties of segmented polyurethane zwitterionomer elastomers. J Macromol Sci B Phys 1984;23:175–99.

[34] Yung LL, Cooper SL. Neutrophil adhesion on phosphorylcholine-containing polyurethanes. Biomaterials 1998;19:31–40. 9678847 10.1016/s0142-9612(97)00220-2

[35] Ishihara K, Ueda T, Nakabayashi N. Preparation of phospholipid polymers and their properties as polymer hydrogel membranes. Polym J 1990;22:355–60.

[36] Ishihara K. Blood-compatible surfaces with phosphorylcholine-based polymers for cardiovascular medical devices. Langmuir 2019;35:1778–87. 30056709 10.1021/acs.langmuir.8b01565

[37] Takami K, Matsuno R, Ishihara K. Synthesis of polyurethanes by polyaddition using diol compounds with methacrylate-derived functional groups. Polymer 2011;52:5445–51.

[38] Ma C, Zhou H, Wu B et al Preparation of polyurethane with zwitterionic side chains and their protein resistance. ACS Appl Mater Interfaces 2011;3:455–61. 21222476 10.1021/am101039q

[39] Jiang S, Cao Z. Ultralow‐fouling, functionalizable, and hydrolyzable zwitterionic materials and their derivatives for biological applications. Adv Mater 2010;22:920–32. 20217815 10.1002/adma.200901407

[40] Mi L, Jiang S. Integrated antimicrobial and nonfouling zwitterionic polymers. Angew Chem Int Ed 2014;53:1746–54. 10.1002/anie.201304060 24446141

[41] Sinclair A, O'Kelly MB, Bai T et al Self-healing zwitterionic microgels as a versatile platform for malleable cell constructs and injectable therapies. Adv Mater 2018;30:1803087. 10.1002/adma.201803087 PMC6588167 30066374

[42] Shao Q, Jiang S. Molecular understanding and design of zwitterionic materials. Adv Mater 2015;27:15–26. 25367090 10.1002/adma.201404059

[43] Chang Y, Chen S, Yu Q et al Development of biocompatible interpenetrating polymer networks containing a sulfobetaine-based polymer and a segmented polyurethane for protein resistance. Biomacromolecules 2007;8:122–7. 17206797 10.1021/bm060739m

[44] Huang C, Li Y, Jiang S. Zwitterionic polymer-based platform with two-layer architecture for ultra low fouling and high protein loading. Anal Chem 2012;84:3440–5. 22409836 10.1021/ac3003769

[45] Hong D, Hung H, Wu K et al Achieving ultralow fouling under ambient conditions *via* surface-initiated ARGET ATRP of carboxybetaine. ACS Appl Mater Interfaces 2017;9:9255–9. 28252277 10.1021/acsami.7b01530

[46] Han X, Hung H, Jain P et al Sterilization, hydration-dehydration and tube fabrication of zwitterionic hydrogels. Biointerphases 2017;12:02C411. 10.1116/1.4983502 PMC5433902 28511543

[47] Yuan H, Qian B, Zhang W et al Protein adsorption resistance of PVP-modified polyurethane film prepared by surface-initiated atom transfer radical polymerization. Appl Surf Sci 2016;363:483–9.

[48] Li P, Luo Z, Li X et al Preparation, evaluation and functionalization of biomimetic block copolymer coatings for potential applications in cardiovascular implants. Appl Surf Sci 2020;502:144085.

[49] Chen H, Zhao Y, Xiong K et al Multifunctional coating based on EPC-specific peptide and phospholipid polymers for potential applications in cardiovascular implants fate. J Mater Chem B 2016;4:7870–81. 32263777 10.1039/c6tb01811d

[50] Cai W, Xiao C, Qian L et al Detecting van der Waals forces between a single polymer repeating unit and a solid surface in high vacuum. Nano Res 2019;12:57–61.

[51] Deng J, Yuan S, Li X et al Heparin/DNA aptamer co-assembled multifunctional catecholamine coating for EPC capture and improved hemocompatibility of vascular devices. Mater Sci Eng C 2017;79:305–14. 10.1016/j.msec.2017.05.057 28629023

[52] Li X, Qiu H, Gao P et al Synergetic coordination and catecholamine chemistry for catalytic generation of nitric oxide on vascular stents. NPG Asia Mater 2018;10:482–96.

[53] Li X, Gao P, Tan J et al Assembly of metal–phenolic/catecholamine networks for synergistically anti-inflammatory, antimicrobial, and anticoagulant coatings. ACS Appl Mater Interfaces 2018;10:40844–53. 30403339 10.1021/acsami.8b14409

[54] Fan Y, Zhang Y, Zhao Q et al Immobilization of nano Cu-MOFs with polydopamine coating for adaptable gasotransmitter generation and copper ion delivery on cardiovascular stents. Biomaterials 2019;204:36–45. 30875517 10.1016/j.biomaterials.2019.03.007

[55] Hao H, Deng Y, Wu Y et al Synthesis of biodegradable waterborne phosphatidylcholine polyurethanes for soft tissue engineering applications. Regen Biomater 2017;4:69–79.

[56] Tan D, Zhang X, Wang J et al Synthesis and phase behavior of polyurethanes end-capped with fluorinated phosphatidylcholine head groups. Chin J Polym Sci 2011;29:615–26.

[57] Tan H, Guo M, Du R et al The effect of fluorinated side chain attached on hard segment on the phase separation and surface topography of polyurethanes. Polymer 2004;45:1647–57.

[58] Wang Z, Yu L, Ding M et al Preparation and rapid degradation of nontoxic biodegradable polyurethanes based on poly (lactic acid)-poly (ethylene glycol)-poly (lactic acid) and l-lysine diisocyanate. Polym Chem 2011;2:601–7.

