# Supplementary material for: Preparation of phospholipid-based polycarbonate urethanes for potential applications of blood-contacting implants
Source: Regen Biomater. 2020 Sep 6;7(5):491–504. doi: 10.1093/rb/rbaa037 (PMC7597807; doi:10.1093/rb/rbaa037)
Supplement: rbaa037_Supplementary_Data [file rbaa037_supplementary_data.docx]

**Supplementary materials**

**Preparation of Phospholipid-Based Polycarbonate Urethanes for Potential Applications of** **Blood-Contacting Implants**

Peichuang Li^a,b^, Wanhao Cai^c^, Xin Li^a,b^, Kebing Wang^a,b^, Lei Zhou^a,b^, Tianxue You^a,b^, Rui Wang^a,b^, Hang Chen^a,b^, Yuancong Zhao^a,b,^*, Jin Wang^a,b,^*, Nan Huang^a,b^

^a^ Key Lab. of Advanced Technology for Materials of Education Ministry, Southwest Jiaotong University, Chengdu 610031, China

^b^ School of Materials Science and Engineering, Southwest Jiaotong University, Chengdu 610031, China

^c^ Institute of Physical Chemistry, University of Freiburg, Albertstraße 21a, 79104 Freiburg, Germany

*Corresponding authors: zhaoyc7320@163.com (Y. Zhao), jinxxwang@263.net (J. Wang).


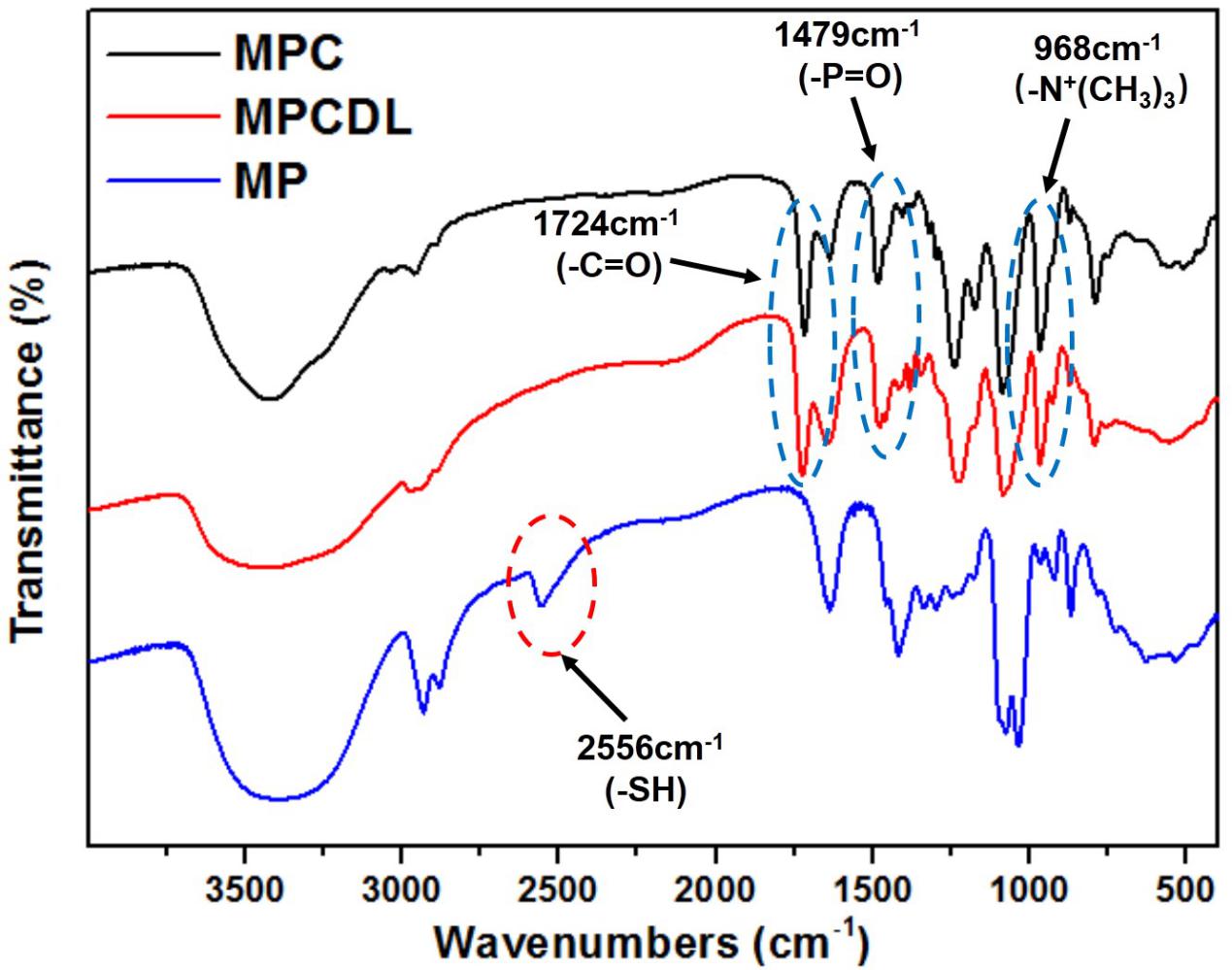


Fig S1. FTIR results of MPCDL.


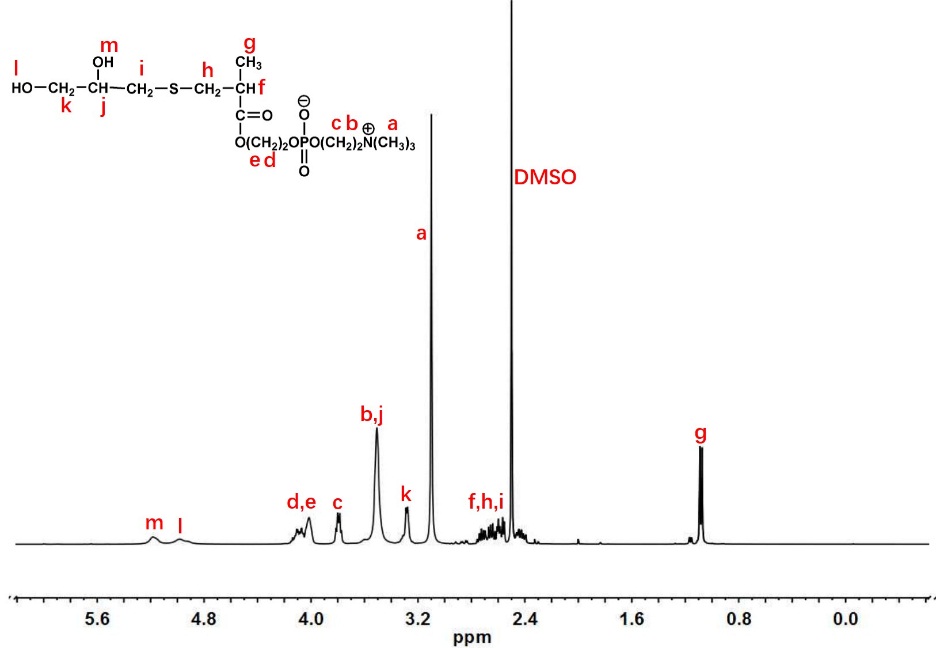


Fig S2. ^1^H NMR result of MPCDL.


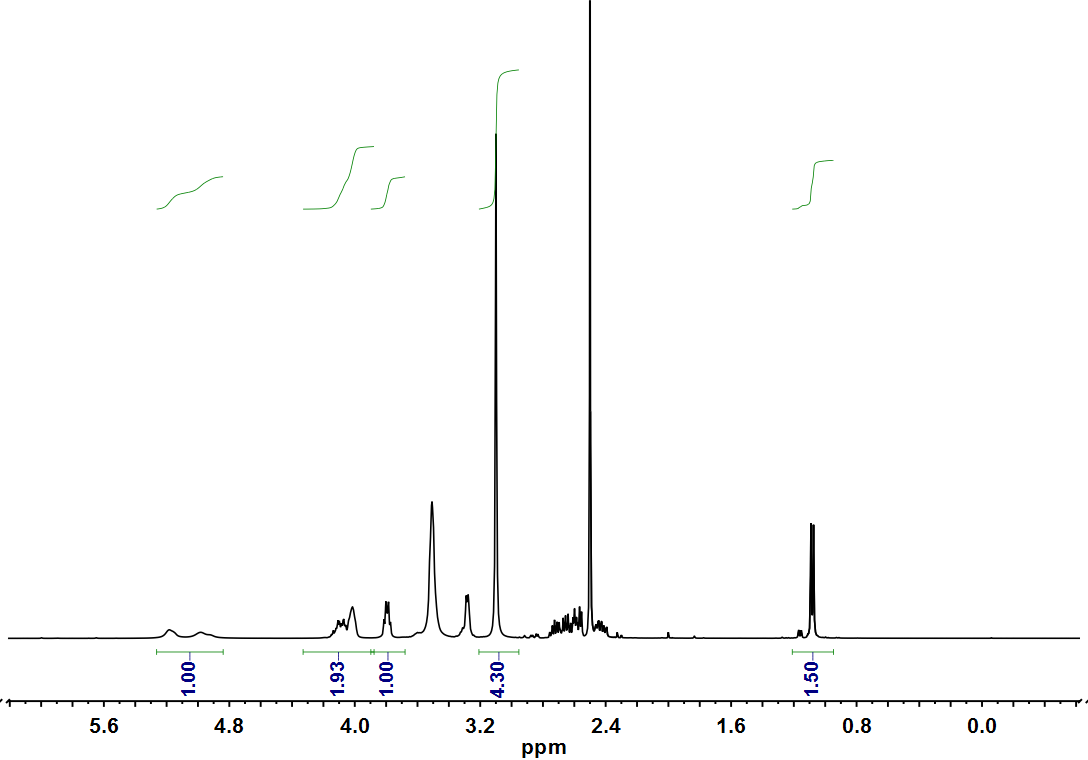


Fig S3. Proton integral result of MPCDL.

Proton integral on NMR spectra of MPCDL was shown in Fig. S3. Because of the effect of deuterium reagent and residual moisture, part of proton integral was not displayed in the Fig. S3. As shown in Fig. S3, the integral ratio of protons in different chemical environments is basically consistent with the theory value.

Fig S4. ESI-MS result of MPCDL.


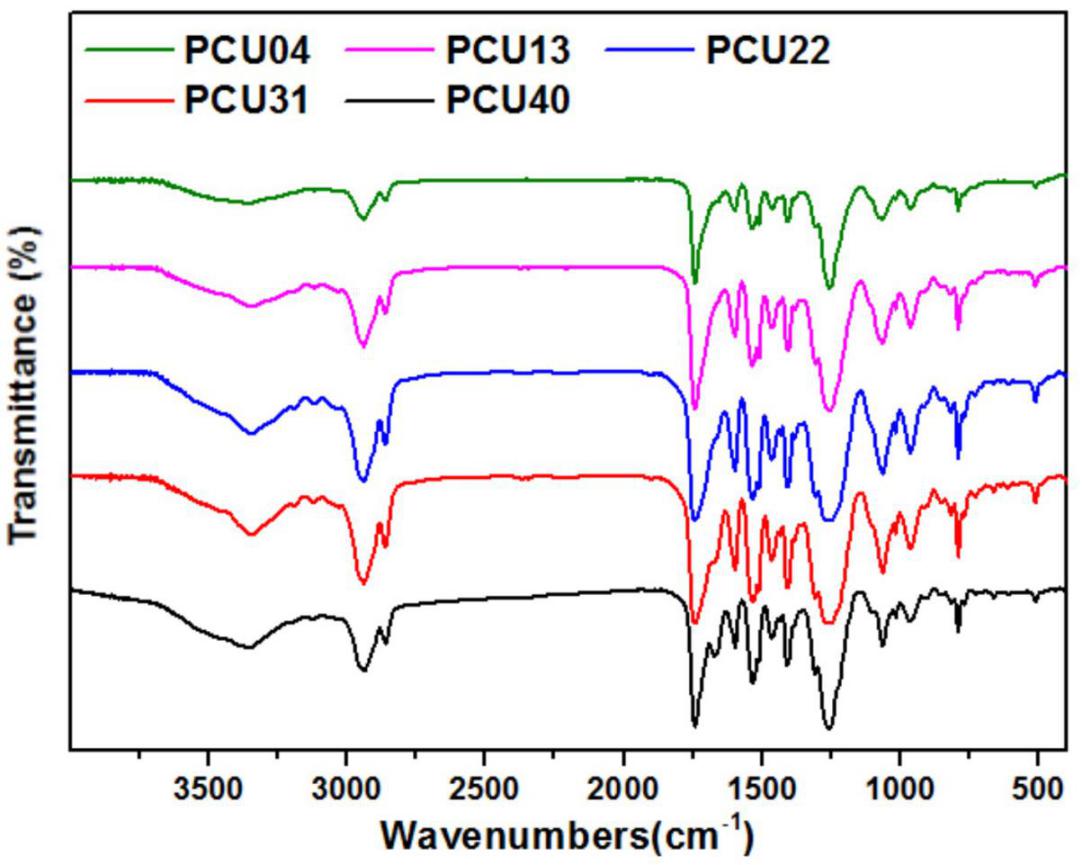


Fig S5. FTIR results of PCUs.

**
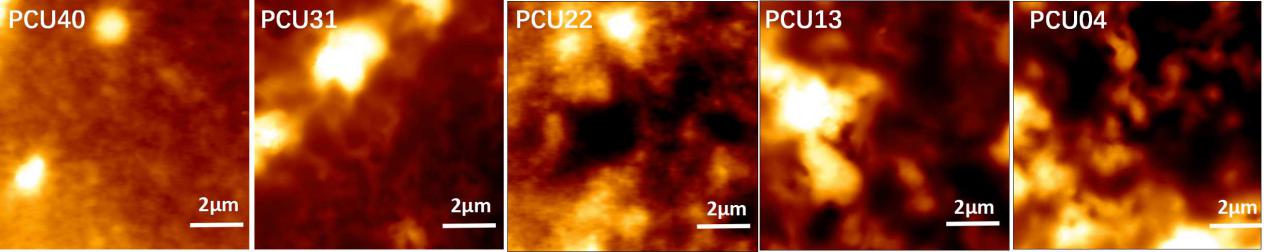
**

Fig S6. AFM images of PCUs films surface.

AFM images of PCUs films surface were shown in Fig. S6. It is found that for PCU40 (without MPCDL), polyurethanes distributed relative uniformly on the surface, indicates a low degree of microphase separation. With the increasing of MPCDL content, more dark and bright areas are observed. These results indicate that the distribution of polyurethanes becomes more nonuniform, i.e., the degree of microphase separation becomes higher [1].

**References**

[1] Tan H, Li J, Guo M *et al*. Phase behavior and hydrogen bonding in biomembrane mimicing polyurethanes with long side chain fluorinated alkyl phosphatidylcholine polar head groups attached to hard block. *Polymer* 2005; **46**: 7230-7239.
